# Barriers to access to care reported by women living with HIV across 27 countries

**DOI:** 10.1080/09540121.2015.1046416

**Published:** 2015-07-14

**Authors:** Margaret Johnson, Anna Samarina, He Xi, José Valdez Ramalho Madruga, Laurent Hocqueloux, Mona Loutfy, Marie-Josée Fournelle, Michael Norton, Jean Van Wyk, Woodie Zachry, Marisol Martinez

**Affiliations:** ^a^ Royal Free Hospital NHS Trust, London,UK; ^b^ Saint-Petersburg City HIV Centre, Petersburg, Russia; ^c^ Guangzhou Eight People's Hospital, Yuexiu District, Guangzhou, China; ^d^ Centro de Referência e Treinamento DST/AIDS, Sã Paulo, Brazil; ^e^ Centre Hospitalier Régional d'Orléans – La Source, Service des Maladies Infectieuses et Tropicales, Orléans, France; ^f^ Women's College Research Institute, University of Toronto, Toronto, Canada; ^g^ AbbVie, Inc., North Chicago, IL, USA

**Keywords:** access to health care, women, HIV, barriers to care, stigma, HIV treatment

## Abstract

Increased access to successful antiretroviral therapy (ART) is necessary in order to achieve an AIDS-free generation. Importantly, slightly over half of the people living with HIV are women. Small studies have described many barriers to accessing treatment and care among women living with HIV. This cross-sectional, non-interventional, epidemiological study assessed the prevalence of barriers to accessing care for women living with HIV across 27 countries, divided into four global regions. HIV-positive women attending routine clinical visits were offered the opportunity to participate in the study. Data describing the study sites and demographic characteristics of the participating women were collected. Participating women filled out questionnaires including the Barriers to Care Scale (BACS) questionnaire, on which they reported the extent to which they found each of the 12 potential barriers to accessing health care problematic. A total of 1931 women living with HIV were included in the study: 760 from Western Europe and Canada (WEC), 532 from Central and Eastern Europe (CEE), 519 from Latin America (LA), and 120 from China. The mean age of participating women was 40.1 ± 11.4 years. A total of 88.2% were currently taking ART. A total of 81.8% obtained HIV treatment under a government health plan. The most prevalent barrier to care was community HIV/AIDS stigma. Community HIV/AIDS knowledge, lack of supportive/understanding work environments, lack of employment opportunities, and personal financial resources were also highly prevalent barriers to accessing care. These findings indicate that, more than 30 years after the start of the AIDS epidemic, stigma is still a major issue for women living with HIV. Continued efforts are needed to improve community education on HIV/AIDS in order to maximize access to health care among women living with HIV.

## Introduction

An estimated 35.3 million people were living with HIV/AIDS worldwide in 2012 (World Health Organization, 2014). Slightly over half of the people living with HIV are women (UNAIDS, 2010). Initiatives working toward an AIDS-free generation aim to prevent mother-to-child transmission, increase access to and uptake of HIV testing, and increase the number of people receiving HIV treatment (US Department of State. United States of America, 2012). Estimates indicate that approximately 14 million people were receiving antiretroviral therapy (ART) in low- and middle-income countries in July 2014 (UNAIDS, 2014b). Increasing this number is important for stemming the AIDS epidemic. The UNAIDS 90:90:90 initiative aims to ensure that by 2020, 90% of the people living with HIV will know their HIV status, 90% of those diagnosed with HIV will receive sustained ART, and 90% of those on ART will have durable viral suppression. Modeling suggests that achieving these goals by 2020 could end the AIDS epidemic by 2030 (UNAIDS, 2014a).

Life expectancy for people living with HIV has increased with the availability of ART (Nakagawa, May, & Phillips, 2013). HIV-positive people on successful ART require ongoing health-care services as they are potentially at increased risk for developing disorders including cardiovascular disease, liver disease, accelerated bone loss, and metabolic disorders (Ali et al., 2014; Amorosa & Tebas, 2006; Deeks, Lewin, & Havlir, 2013). Health issues of particular concern in women living with HIV include depression and neurocognitive impairment, cervical cancer, and early menopause (Loutfy et al., 2013). Women may be reluctant to access non-HIV health-care services due to concerns over disclosure of their HIV status (Turan, Miller, Bukusi, Sande, & Cohen, 2008).

Clinical outcomes may be poorer for women living with HIV than for men (Aziz & Smith, 2012; Meyer et al., 2014). The higher incidence of mental health-related issues, which can complicate HIV/AIDS care in HIV-positive women compared to men, contributes to this disparity (Aziz & Smith, 2012; Robertson et al., 2014). Women may be less likely to receive ART than men (Gebo et al., 2005). Barriers to accessing care that disproportionately affect women include transportation, lack of gender autonomy, health-care systems, stigma, economic constraints, lack of knowledge, and gender roles (Arrivillaga-Quintero, 2010; Donahue, Dube, Dow, Umar, & Van Rie, 2012; Duff, Kipp, Wild, Rubaale, & Okech-Ojony, 2010; Heckman et al., 1998; Moneyham et al., 2010; Sarnquist et al., 2011; Stevens & Keigher, 2009). Studies identifying these barriers to accessing care in women have generally been limited in size (40–226 patients) and geographic scope.

The identification of barriers to accessing care for women living with HIV could help indicate the types of initiatives needed to maximize access to care for women. The ELLA (a cross-sectional, multi-country, non-interventional EpidemioLogical study to investigate the popuLation and disease characteristics, barriers to care and quAlity of life for women living with HIV) study aimed to identify self-reported barriers to care experienced by women living with HIV who have navigated those barriers and are receiving health care.

## Methods

### Study population

This was a cross-sectional, multi-country, non-interventional epidemiological study. Women from 114 sites in 27 countries were included (Table 1). The geographic regions represented were: Western Europe and Canada (WEC), Central and Eastern Europe (CEE), Latin America (LA), and Asia (China). Sites known to provide treatment to women with HIV were selected. Patients were enrolled from July 2012 to September 2013. Eligible patients were HIV-1 positive females ≥& >18 years of age who were diagnosed with HIV infection ≥3 months prior to study inclusion.

**Table 1.** Geographical disposition of patients.

Region	County	Number of sites	Number of patients enrolled
Global *(total)*		114	1931
WEC *(total)*		74	760
	Austria	3	18
	Canada	6	72
	France	8	82
	Germany	4	70
	Greece	4	40
	Ireland	1	20
	Israel	3	37
	Italy	6	99
	Netherlands	1	25
	Norway	3	21
	Portugal	6	93
	Spain	16	87
	Sweden	4	25
	Switzerland	2	27
	UK	7	44
CEE *(total)*		20	532
	Czech Republic	3	20
	Estonia	3	99
	Romania	8	200
	Russia	5	171
	Slovenia	1	42
LA *(total)*		17	519
	Argentina	3	101
	Brazil	1	90
	Chile	1	50
	Columbia	6	84
	Mexico	2	115
	Venezuela	4	79
Asia *(total)*		3	120
	China	3	120

### Procedures

Women living with HIV attending a routine clinic visit were invited to participate, and were enrolled using a non-random sequential sampling frame. Women provided written authorization for use and/or disclosure of anonymized health data (and written informed consent, where applicable). Reasons for declining participation were captured. Each site completed a form capturing data about the site, services available, and care guidelines for HIV-1 infected patients including women's services. The investigators were physicians at the sites who received training from the sponsor. The site research coordinators completed a form reporting demographics, social and educational background, HIV infection-related data, relevant medical history, and health-care utilization of each participating woman. Women were asked to complete four questionnaires: Barriers to Care Scale (BACS; Heckman et al., 1998), Reproductive Choices (NIAID AIDS Clinical Trials Group), Overall Health Status Assessment (NIAID AIDS Clinical Trials Group), and Symptoms Distress Module (NIAID Adult AIDS Clinical Trials Group).

### Measures

The BACS is a 12-item self-administered scale, through which HIV-positive individuals indicate the severity of various barriers to care and service provision (Heckman et al., 1998). The 12 BACS items are: (1) long distance to medical facilities/personnel, (2) decline to provide direct care to persons with HIV/AIDS, (3) lack of trained and competent health-care providers in AIDS care, (4) lack of transportation, (5) lack of mental health health-care provider, (6) lack of psychological support, (7) community HIV/AIDS knowledge, (8) community HIV/AIDS stigma, (9) lack of employment opportunities, (10) lack of supportive/understanding work environments, (11) personal financial resources, and (12) lack of adequate/affordable housing. The BACS comprises four sub-scales: (1) geography/distance barriers (BACS items 1 and 4), (2) medical and psychological service barriers (BACS items 2, 3, 5, and 6), (3) community stigma barriers (BACS items 7 and 8), and (4) personal resource barriers (BACS items 9, 10, 11, and 12) (Heckman et al., 1998). For each of the 12 items, respondents use a 4-point Likert scale (1 = No problem at all, 2 = Very slight problem, 3 = Somewhat of a problem, 4 = Major problem) to indicate the extent to which each listed barrier made it difficult for them to receive the care, services, or opportunities they wished to obtain (Heckman et al., 1998). In this study, a score ≥2 was considered significant.

### Statistical analysis

The primary endpoint was the prevalence of barriers to accessing health care for women living with HIV globally and by geographic region. Women were subdivided into regions for analysis due to geography and health-care system similarities within the regions. Barriers to health-care index (average number of barriers that women living with HIV indicated were problematic, regardless of the reported extent of the problem) and BACS severity scores (average response on the Likert scale) were also calculated (Full definitions in Table 2). Only women with no missing data on the 12 BACS items were included in the barriers to health-care index analysis. Women were excluded from overall or sub-scale BACS severity score analysis if they were missing >50% of all BACS items or a sub-scale's items, respectively. For women with ≤50% of items missing, the mean substitution method was used to impute the missing items. Mean score (standard deviation) was calculated for the barriers to health-care index and BACS severity scores for the global population and each geographic region. The demographic constitution, reproductive choices, and HIV disease characteristics of the global population and of women in each region were summarized descriptively.

**Table 2.** Measures of barriers to accessing health care.

Measure	Definition	Range of possible scores
Prevalence of barriers to accessing health care	Proportion of women living with HIV responding in each of the 4 response categories (1 = No problem at all, 2 = Veryslight problem, 3 = Somewhat of a problem, 4 = Major problem) for each BACS item	0–100%
Barriers to health-care index	The average number of barriers that women living with HIV indicated were problematic, regardless of the reported extent of the problem (response category 2, 3, or 4)	0–12
BACS-severity score	A measure of problem severity, calculated as the average response on the Likert scale. BACS-severity scores were calculated for each BACS item, for the BACS questionnaire overall, and for the four sub-scales. Higher scores indicate increased problem severity. Scores ≥2 were considered significant.	1–4

A univariate multilevel analysis of variance (ANOVA) tested association of variables with increased BACS severity scores. Variables associated with increased BACS severity scores at a significance level of 20% in the univariate analysis were candidates to be entered in a multivariate multilevel ANOVA. The multivariate multilevel ANOVA determined factors that were significant at 5% while controlling for other significant covariates. The stepwise selection method was used to enter variables into and remove variables from the model (significance level of 0.15 for entering/removing variables). To arrive at the final model, interaction terms between the variable indicating the geographic area of patients and each significant factor were tested to assess whether the effect of the factor on the BACS severity score was different between geographic areas. Pairwise comparisons of BACS severity score among geographic areas were performed.

## Results

### Patient characteristics

A total of 1931 women living with HIV were included in the study: 760 from WEC, 532 from CEE, 519 from LA, and 120 from China. A total of 563 women declined to participate. The most common main reasons for not participating were “no time” for 261 women (46.4%) and “not interested” for 151 women (26.8%). Demographic and disease characteristics of participating women are presented in Table 3. In the global population, mean age was 40.1 ± 11.4 years. The most common risk factor for acquiring HIV was sexual contact (83.0% of the global population). Blood transfusion or organ transplant was a risk factor for acquiring HIV in 25.0% of women from China. Overall, 57.9% of women had been living with HIV for >5 years. A total of 78.8% of the women were non-immigrants, 82.9% were living in urban areas, and 40.4% were legally married. Among the 1067 (55.3%) women living with a partner, 45.6% reported that their partners were HIV-positive. In the global population, 33.5% of women had >12 years of formal education; 36.0% of participants were unemployed.

**Table 3.** Characteristics of women living with HIV in the ELLA study.

	Global *N* = 1931	WEC *N* = 760	CEE *N* = 532	LA *N* = 519	China *N* = 120
Mean age (years)	40.1 ± 11.4	44.0 ± 10.8	33.2 ± 9.8	42.2 ± 11.1	37.7 ± 8.4
*Risk factor for acquiring HIV*					
Sexual contact	1602 (83.0)	647 (85.1)	371 (69.7)	502 (96.7)	82 (68.3)
Intravenous drug user	150 (7.8)	79 (10.4)	63 (11.8)	4 (0.8)	4 (3.3)
Blood transfusion or organ transplant	126 (6.5)	20 (2.6)	66 (12.4)	10 (1.9)	30 (25.0)
Mother to child transmission	20 (1.0)	11 (1.4)	4 (0.8)	3 (0.6)	2 (1.7)
Other	2 (0.1)	1 (0.1)	–	–	1 (0.8)
Unknown	31 (1.6)	2 (0.3)	28 (5.3)	–	1 (0.8)
*Immigration status*					
Not immigrant	1521 (78.8)	436 (57.4)	507 (95.3)	458 (88.2)	120 (100)
Immigrant for <1 year	7 (0.4)	3 (0.4)	–	4 (0.8)	–
Immigrant for 1–5 years	64 (3.3)	56 (7.4)	4 (0.8)	4 (0.8)	–
Immigrant for >5 years	339 (17.6)	265 (34.9)	53 (10.2)	53 (10.2)	–
*Residence*					
Rural area	330 (17.1)	134 (17.6)	137 (25.8)	26 (5.0)	33 (27.5)
Urban area	1601 (82.9)	626 (82.4)	395 (74.2)	493 (95.0)	87 (72.5)
*Legally married*					
No	1151 (59.6)	460 (60.5)	321 (60.3)	347 (66.9)	23 (19.2)
Yes	780 (40.4)	300 (39.5)	211 (39.7)	172 (33.1)	97 (80.8)
*Living status*					
Living alone	415 (21.5)	223 (29.3)	96 (18.0)	71 (13.7)	25 (20.8)
Not living alone	1516 (78.5)	537 (70.7)	436 (82.0)	448 (86.3)	95 (79.2)
*Partner HIV status for women living with partner*					
HIV-negative	513 (48.1)	226 (59.5)	149 (40.8)	93 (39.1)	45 (53.6)
HIV-positive	487 (45.6)	134 (35.3)	191 (52.3)	128 (53.8)	34 (40.5)
Unknown	67 (6.3)	20 (5.3)	25 (6.8)	17 (7.1)	5 (6.0)
*Regular support from family or friends*					
No	770 (39.9)	373 (49.1)	123 (23.1)	222 (42.8)	52 (43.3)
Yes	1161 (60.1)	387 (50.9)	409 (76.9)	297 (57.2)	68 (56.7)
*Disclosure of HIV status*					
No disclosure or disclosed to intimate relations	1686 (87.3)	643 (84.6)	477 (89.7)	450 (86.7)	116 (96.7)
Complete or extended disclosure	243 (12.6)	115 (15.1)	55 (10.3)	69 (13.3)	4 (3.3)
Missing	2 (0.1)	2 (0.3)	–	–	–
*Payment method for HIV treatment*					
Government or private insurance	1774 (91.9)	742 (97.6)	529 (99.4)	471 (90.8)	32 (26.7)
Combination of government and/or private and/or out-of-pocket	61 (3.2)	6 (0.8)	–	–	55 (45.8)
Self-pay, out-of-pocket	92 (4.8)	11 (1.4)	1 (0.2)	47 (9.1)	33 (27.5)
Not available	4 (0.2)	1 (0.1)	2 (0.4)	1 (0.2)	–
*Years of formal education completed*					
≤12	1256 (65.0)	429 (56.4)	356 (66.9)	375 (72.3)	96 (80.0)
>12	647 (33.5)	304 (40.0)	176 (33.1)	143 (27.6)	24 (20.0)
Not available	28 (1.5)	27 (3.6)	0	1 (0.2)	0
*Current primary occupation*					
Employed/self-employed	1014 (52.5)	399 (52.5)	253 (47.6)	287 (55.3)	75 (62.5)
Retired	157 (8.1)	79 (10.4)	31 (5.8)	44 (8.5)	3 (2.5)
Student	64 (3.3)	34 (4.5)	19 (3.6)	11 (2.1)	–
Unemployed	696 (36.0)	248 (32.6)	229 (43.0)	177 (34.1)	42 (35.0)
Time unemployed (months)					
≤12 months	114 (5.9)	39 (5.1)	33 (6.2)	35 (6.7)	7 (5.8)
>12 months	582 (30.1)	209 (27.5)	196 (36.8)	142 (27.4)	35 (29.2)
Mean number of children*	1.4 ± 1.4	1.4 ± 1.4	0.9 ± 0.9	1.9 ± 1.6	1.0 ± 1.0
*Time from HIV diagnosis to enrollment (years)*					
<1	118 (6.1)	20 (2.6)	29 (5.5)	37 (7.1)	32 (26.7)
1–5	617 (32.0)	171 (22.5)	214 (40.2)	173 (33.3)	59 (49.2)
>5–10	485 (25.1)	170 (22.4)	154 (29.0)	142 (27.4)	19 (15.8)
>10	633 (32.8)	359 (47.2)	129 (24.3)	140 (27.0)	5 (4.2)
Unknown	78 (4.0)	40 (5.3)	6 (1.1)	27 (5.2)	5 (4.2)
*Use of antiretroviral therapy (ART)*					
Never used ART	157 (8.1)	34 (4.5)	64 (12.0)	42 (8.1)	17 (14.2)
Used or currently using ART	1774 (91.9)	726 (95.5)	468 (88.0)	477 (91.9)	103 (85.8)
*Last viral load (copies/mL)*					
<400	1277 (66.1)	631 (83.0)	272 (51.1)	317 (61.1)	57 (47.5)
≥400	361 (18.7)	70 (9.2)	189 (35.3)	91 (17.5)	11 (9.2)
Unknown	293 (15.2)	59 (7.8)	71 (13.3)	111 (21.4)	52 (43.3)
*Last CD4 + (cells/mm^3^)*					
<200	209 (10.8)	48 (6.3)	75 (14.1)	65 (12.5)	21 (17.5)
201–350	286 (14.8)	88 (11.6)	96 (18.0)	81 (15.6)	21 (17.5)
351–500	408 (21.1)	136 (17.9)	143 (26.9)	92 (17.7)	37 (30.8)
>500	982 (50.9)	487 (64.1)	215 (40.4)	249 (48.0)	31 (25.8)
Unknown	46 (2.4)	1 (0.1)	3 (0.6)	32 (6.2)	10 (8.3)

Plus-minus values are means ± SD.

*For mean number of children, *N* = 119 for China, *N* = 531 for CEE, *N* = 512 for LA, *N* = 744 for WEC, and *N* = 1906 for the global population.

In the global population, 91.9% of women had used or were currently using ART, reflecting wide ART use in all geographic regions. In total, 18.7% of women had a most recent recorded viral load ≥400 copies/mL, and 10.8% had a most recent recorded CD4 count <200 cells/mm^3^.

### Prevalence of barriers to care

**Figure 1.**
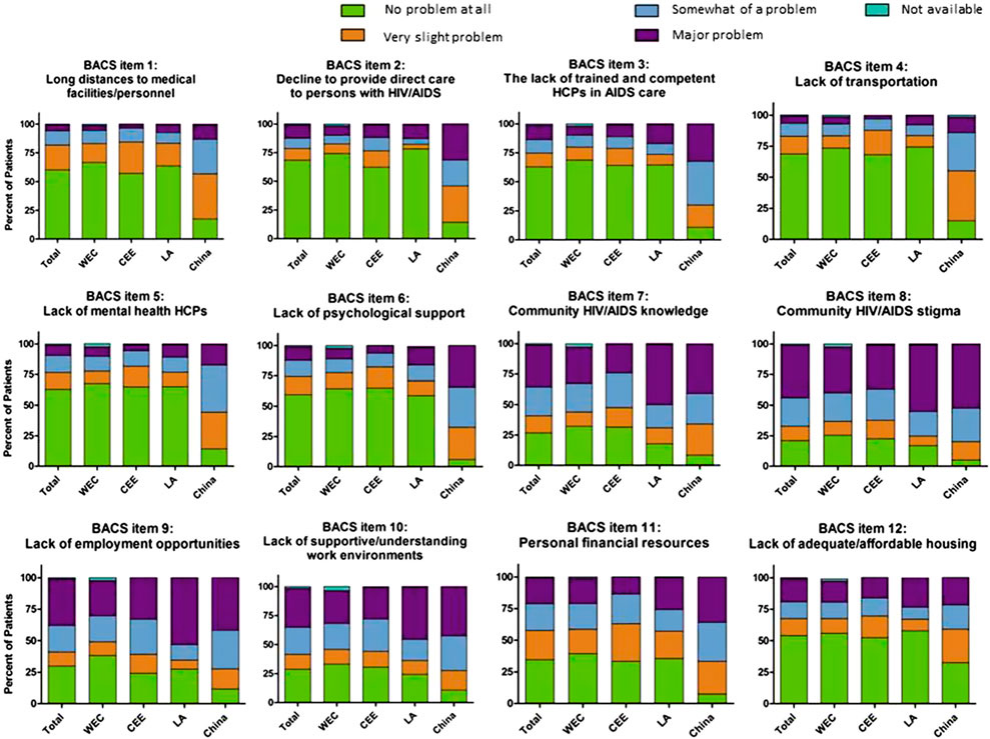
Prevalence of barriers to health care. Proportion of women responding that potential barriers included in the BACS questionnaire were no problem at all, a very slight problem, somewhat of a problem, or a major problem.

The prevalence of each of the 12 barriers to health care in the BACS questionnaire is in Figure 1. Globally and by region, community HIV/AIDS stigma was the most prevalent barrier for women in this study; 77.7% of women in the global population identified this barrier as problematic, regardless of severity. Also highly prevalent were the barriers of community HIV/AIDS knowledge, lack of supportive/understanding work environments, lack of employment opportunities, and personal financial resources, which were identified as problematic (regardless of severity) by 72.1%, 69.2%, 69.7%, and 64.8% of the global population, respectively. Among the four regions, women in China reported the highest prevalence of barriers to health care over all the 12 BACS items.

### Barriers to health-care index

For the global population, the mean barriers to health-care index was 6.2 ± 3.5, indicating that women in the study identified an average of 6.2 of the 12 barriers as problematic, regardless of severity. The mean barriers to health-care index was 5.4 ± 3.5 for WEC, 6.2 ± 3.2 for CEE, 6.1 ± 3.1 for LA, and 10.9 ± 1.9 for China.

**Figure 2.**
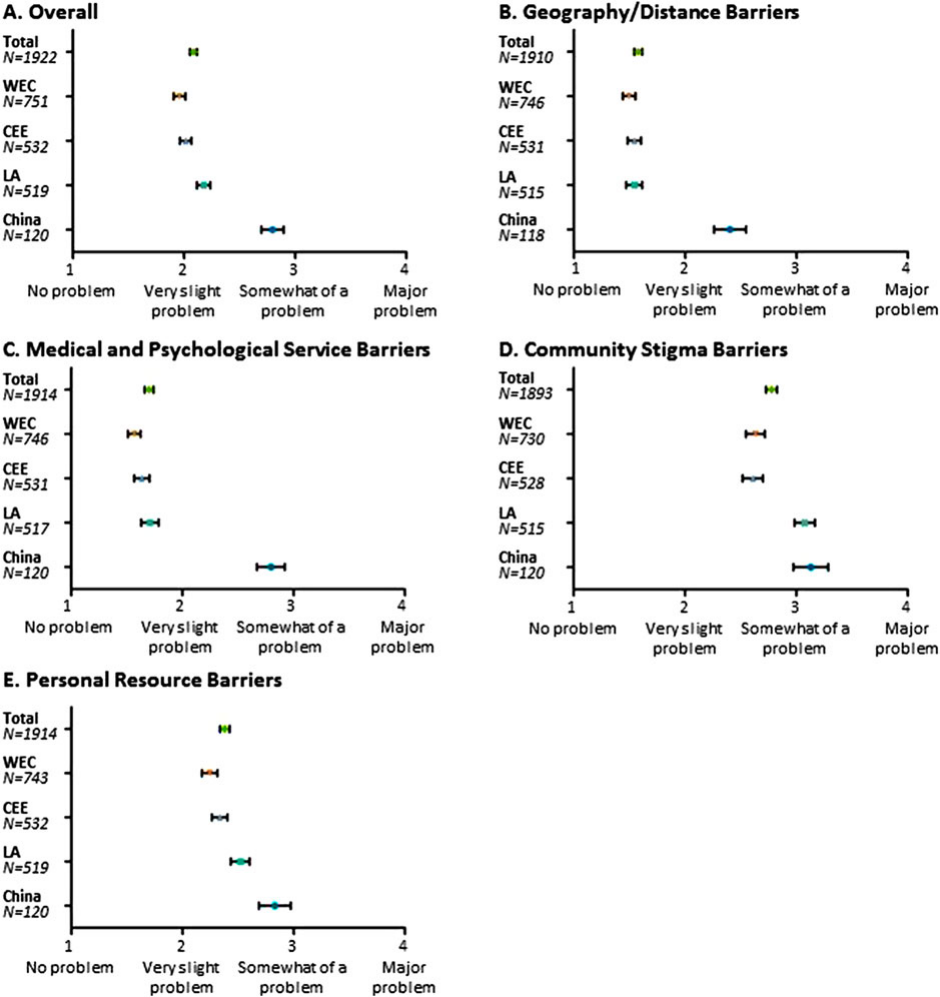
BACS severity scores. The position of the circle indicates the mean BACS severity score; the bars are 95% confidence intervals. Scores ≥2 were considered significant. The geography/distance subscale comprises BACS items 1 and 4. The medical and psychological service barriers subscale comprises BACS items 2, 3, 5, and 6. The community stigma barriers subscale comprises BACS items 7 and 8. The personal resource barriers subscale comprises BACS items 9, 10, 11, and 12.

Overall for the 12-item BACS questionnaire, the BACS-severity score was 2.1 ± 0.7 for the global population, with scores of ≥2.0 considered significant (Figure 2). The mean scores were 2.0 ± 0.7 for WEC, 2.0 ± 0.6 for CEE, 2.2 ± 0.7 for LA, and 2.8 ± 0.6 for China.

For the global population, mean sub-scale BACS severity scores were 1.6 ± 0.8, 1.7 ± 0.9, 2.8 ± 1.1, and 2.4 ± 0.9 for geography/distance, medical and psychological service, community stigma, and personal resource barriers, respectively (Figure 2). For the global population and each region, mean scores were numerically highest for community stigma barriers. For community stigma barriers, LA and China had a higher mean score (3.1 for each) compared to other regions.

BACS severity scores for each BACS item are given in Table 4. In the global population, the highest mean BACS severity scores were for community residents’ stigma against persons living with HIV/AIDS (2.9 ± 1.2), community HIV/AIDS knowledge (2.7 ± 1.2), lack of employment opportunities (2.7 ± 1.3), lack of supportive/understanding work environments (2.6 ± 1.2), personal financial resources (2.3 ± 1.1), and lack of adequate/affordable housing (2.0 ± 1.2). Mean BACS severity score for all other items were <2.0.

**Table 4.** BACS severity scores for the 12 BACS items.

BACS Item	Global	WEC	CEE	LA	China
*BACS item 1: Long distances to medical facilities/personnel*	1.6 ± 0.9 *N* = 1919	1.5 ± 0.9 *N* = 752	1.6 ± 0.8 *N* = 531	1.6 ± 0.9 *N* = 517	2.4 ± 0.9 *N* = 119
*BACS item 2: Decline to provide direct care to persons with HIV/AIDS*	1.6 ± 1.1 *N* = 1914	1.5 ± 1.0 *N* = 748	1.7 ± 1.1 *N* = 529	1.5 ± 1.0 *N* = 517	2.7 ± 1.1 *N* = 120
*BACS item 3: The lack of trained and competent HCPs in AIDS care*	1.7 ± 1.1 *N* = 1909	1.6 ± 1.0 *N* = 744	1.7 ± 1.0 *N* = 528	1.8 ± 1.2 *N* = 517	2.9 ± 1.0 *N* = 120
*BACS item 4: Lack of transportation*	1.5 ± 0.9 *N* = 1916	1.5 ± 0.9 *N* = 749	1.5 ± 0.8 *N* = 532	1.5 ± 0.9 *N* = 517	2.4 ± 0.9 *N* = 118
*BACS item 5: Lack of mental health HCPs*	1.7 ± 1.0 *N* = 1905	1.6 ± 1.0 *N* = 740	1.6 ± 0.9 *N* = 528	1.7 ± 1.1 *N* = 517	2.6 ± 0.9 *N* = 120
*BACS item 6: Lack of psychological support*	1.8 ± 1.1 *N* = 1909	1.6 ± 1.0 *N* = 742	1.6 ± 0.9 *N* = 531	1.8 ± 1.1 *N* = 516	3.0 ± 0.9 *N* = 120
*BACS item 7: Community HIV/AIDS knowledge*	2.7 ± 1.2 *N* = 1907	2.5 ± 1.2 *N* = 738	2.5 ± 1.2 *N* = 532	3.0 ± 1.2 *N* = 517	3.0 ± 1.0 *N* = 120
*BACS item 8: Community HIV/AIDS stigma*	2.9 ± 1.2 *N* = 1905	2.7 ± 1.2 *N* = 741	2.9 ± 1.2 *N* = 528	3.1 ± 1.1 *N* = 516	3.3 ± 0.9 *N* = 120
*BACS item 9: Lack of employment opportunities*	2.7 ± 1.3 *N* = 1912	2.4 ± 1.3 *N* = 742	2.7 ± 1.2 *N* = 532	2.9 ± 1.3 *N* = 518	3.0 ± 1.0 *N* = 120
*BACS item 10: Lack of supportive/understanding work environments*	2.6 ± 1.2 *N* = 1899	2.5 ± 1.2 *N* = 732	2.5 ± 1.2 *N* = 530	2.8 ± 1.2 *N* = 517	3.0 ± 1.0 *N* = 120
*BACS item 11: Personal financial resources*	2.3 ± 1.1 *N* = 1920	2.2 ± 1.2 *N* = 752	2.2 ± 1.0 *N* = 532	2.3 ± 1.2 *N* = 516	3.0 ± 1.0 *N* = 120
*BACS item 12: Lack of adequate/affordable housing*	2.0 ± 1.2 *N* = 1918	1.9 ± 1.2 *N* = 747	1.9 ± 1.1 *N* = 532	2.0 ± 1.3*N* = 519	2.3 ± 1.1 *N* = 120

Mean ± SD is shown. A score of 1 = No problem at all, 2 = Very slight problem, 3 = Somewhat of a problem, and 4 = Major problem.

### Factors associated with overall BACS severity scores

Multivariate multilevel ANOVA determined factors associated with overall BACS severity scores (Table 5). Significantly associated factors were residence in China, younger age, unemployment, self-pay for care, presence of comorbidities, smoking, changing treatment facilities in the previous 12 months, missing a scheduled appointment, sites not adhering to treatment guidelines, non-availability of contraceptive agents, and the availability of routine human papillomavirus (HPV) screening.

**Table 5.** Factors associated with overall BACS severity score in multivariate analysis.

			Multivariate analysis	
Factor	BACS-severity score	Comparator	Estimate	*P* value
*Region*				
CEE	2.02 ± 0.61	WEC	–0.1342	0.1308
China	2.80 ± 0.55	WEC	0.7204	**0.0005**
LA	2.18 ± 0.71	WEC	0.0827	0.3332
WEC	1.96 ± 0.72			
*Age (y)*				
< 50	2.12 ± 0.71	50 and above	0.1377	**0.0015**
≥50	1.94 ± 0.68			
*Unemployment*				
Yes	2.20 ± 0.72	No	0.1292	**0.0005**
No	2.02 ± 0.70			
*Payment plan HIV treatment*				
Self-pay, out of pocket	2.75 ± 0.63	Government or private insurance	0.2544	**0.0357**
Government or private insurance	2.03 ± 0.69			
*Number of comorbidities*				
1	2.08 ± 0.70	None	0.1050	**0.0084**
2	2.11 ± 0.70	None	0.1077	**0.0399**
≥3	2.18 ± 0.71	None	0.2956	**<0.0001**
None	2.06 ± 0.72			
*Smoking history*				
Smoker	2.12 ± 0.69	Never or ex-smoker	0.1041	**0.0044**
Never or ex-smoker	2.07 ± 0.72			
*Changed treatment facility during last 12 months*				
Yes	2.31 ± 0.80	No	0.2450	**0.0058**
No	2.07 ± 0.70			
*Missed scheduled appointments*				
Yes	2.16 ± 0.72	No	0.1079	**0.0218**
No	2.07 ± 0.71			
*Adherence to reported guidelines*				
Yes	2.05 ± 0.70	No	–0.0494	0.4335
No	2.13 ± 0.73			
*Contraceptive drug*				
Available	2.01 ± 0.71	Not available	–0.1655	**0.0150**
Not available	2.16 ± 0.70			
*HPV test routine*				
No	2.01 ± 0.70	Yes	–0.2229	**0.0006**
Yes	2.17 ± 0.71			

Other factors tested but not retained in the final model were immigration status (immigrant, non-immigrant), residence (rural, urban), living status (alone, not alone), partner/husband HIV status (negative, positive), regular family friends support (no, yes), disclosure of HIV status (no disclosure or disclosed to close relations, complete or extended disclosure), years of formal education (≤12, >12), number of children that the woman has had (1, 2, 3, 4, 5, none), number of children under 18 years at home (≥1, none), last viral load (<400 copies /mL, ≥400 copies/mL), last recorded CD4 (<200 cells/mm^3^, 200–350 cells/mm^3^, 351–500 cells/mm^3^, > 500 cells/mm^3^), risk factor of acquiring HIV (blood transfusion or organ transplant, intravenous drug user, mother-to-child transmission, sexual contact), time from HIV diagnosis to enrollment (<1 year, 1–5 years, >5–10 years, >10 years), antiretroviral therapy use (never used, previously used or currently using), diagnosed with AIDS-defining illness (yes, no), type of site (private health services, national health services), obstetrics/gynecology/pediatric/contraceptive clinic services (available, not available), psychologist (available, not available), childcare and transportation service (available, not available), mental health-related drug (available, not available), hormonal replacement (available, not available), none female therapies (no, yes), average visit frequency (once a year, more than once a year), frequency of mental health disorder assessment (assessed, not assessed).

## Discussion

The current study represents a large and diverse assessment of barriers to accessing health care in 1931 women living with HIV in four distinct regions. Although women included in this study were all accessing care and a large portion of them had been infected for >5 years, community HIV/AIDS stigma was identified as a significant barrier to accessing care, as observed in previous studies (Cavaleri et al., 2010; Heckman et al., 1998). Across regions, 36.1–54.5% of women reported that community HIV/AIDS stigma was a major problem when accessing care. In addition, major problems were reported for community HIV/AIDS knowledge by 24.2–49.7% of women, lack of supportive/understanding work environments by 27.6–45.3% of women, lack of employment opportunities by 28.0–53.0% of women, and personal financial resources by 13.5–35.8% of women. Long distances to medical facilities/personnel were reportedly a major problem for 3.4–12.5% of women, indicating the presence of geographic barriers. The lack of trained and competent health-care providers in AIDS care was reported as a major barrier in 7.8–32.5% of women, highlighting the presence of medical service barriers.

Multivariate analysis indicated several factors associated with increased barrier severity in the global population. Unemployment and out-of-pocket payment for HIV treatment were associated with increased barrier severity, consistent with the identification of financial barriers to accessing care in this study and a small previous study of women living with HIV in the USA (Moneyham et al., 2010). Presence of comorbidities was also associated with increased barrier severity in the current analysis, corresponding with a previous study reporting that not feeling well enough to attend visits is a barrier to accessing care in HIV-positive women in the USA (Cavaleri et al., 2010). Changing treatment facility during the last 12 months and missed scheduled appointments were also associated with increased barrier severity in this analysis. The association of these factors with barrier severity reflects the inconsistency of treatment that may result from high barriers to accessing care. China, which had the highest BACS severity scores of any region, also had the highest proportion of women whose time since diagnosis was <1 year. However, the time since HIV diagnosis was not identified as a factor associated with barrier severity. Future analyses of data from this study will explore the associations of the level of HIV status disclosure with compliance with general women's health-care services, availability of other health-care services (i.e., obstetrics/gynecologic and mental health services) at the primary care site with accessing these health-care services, and the level of HIV status disclosure with the severity and prevalence of stigma.

Among women in this study, few disclosed their HIV status beyond their intimate relations. Stigma was perceived as a major problem in accessing care. Interestingly, despite years of HIV awareness, the severity scores reported for HIV/AIDS stigma were comparable to those reported in a 1998 study of people living with HIV in the USA (Heckman et al., 1998). The reported stigma may be from the community or self-perceived. Stigma may lead to missed appointments or reluctance to access other needed health-care services outside the primary care facility. When referring a woman living with HIV to other health-care providers, primary care physicians may need to employ additional support services to ensure the woman is comfortable with visiting the new provider and disclosing her HIV status. Elimination of stigma is of great importance to the global community. Removal of this barrier would lead to greater access to health care by women living with HIV, and thereby decreased HIV/AIDS mortality and decreased HIV transmission. Despite declines in AIDS cases and HIV/AIDS mortality, current reports indicate that HIV incidence remains the same in some subpopulations (Maartens, Celum, & Lewin, 2014). Community knowledge is a cornerstone of prevention. Results of this study reinforce the need to continue efforts to educate the general community and health-care providers on HIV to lessen stigma, increase disclosure, and decrease worldwide incidence of HIV.

This study has several limitations. Patient ethnicity was not recorded. The site selection was by invitation and voluntary and not intended to ensure a balance of sites with different levels of services offered. All participating women were attending site visits; correspondingly, a high proportion (91.9%) of women in the study had used or were currently using ART. This study was designed to identify barriers that were significant even in women able to reach to services. Women not visiting health-care facilities likely experience additional barriers to accessing care. Furthermore, different countries were grouped by geographic regions, but the characteristics of the HIV epidemic and income level of the individuals may vary within the region. This heterogeneity may produce bias in analysis. China was the only Asian country included, which may limit the applicability of the results to the entire region. Africa, which has the highest number of HIV-positive women, was not represented in this study.

An additional study limitation is that the BACS questionnaire has only been validated in HIV-positive patients in the USA, and this study enrolled patients in other regions. All questionnaires had dual translation (forward and backward); therefore, language barriers or misinterpretation was expected to be minimal. Women with analphabetism, who likely experience significant barriers to accessing health care, were not included in this study. Furthermore, there was no comparison with men living with HIV.

Each of the 12 potential barriers to health care explored in this study had a higher prevalence in patients from China compared to other regions. Notably, there were only 120 participants from China, which limits the interpretation of these data. The Chinese women in this study came from three sites, all of which were in large urban areas. Like the women from other regions, women from China were navigating barriers and receiving care. The general population of HIV-positive women in China would be expected to encounter additional barriers to accessing care.

Despite China's National Free Antiretroviral Treatment Program, 27.5% of women in this country reported self-pay for HIV treatment. This reflects the ineligibility of migrant workers for this program. Also, this program covers payment for specific antiretroviral drugs; women reporting self-pay may be receiving drugs not covered by this program.

This large epidemiologic study demonstrates that despite significant advances in health-care services and 30 years of HIV awareness, HIV-positive women still experience significant barriers to accessing care. The high prevalence of community HIV/AIDS stigma both in the global population and in each region indicates that continued efforts are needed to improve community education on HIV/AIDS in order to maximize access to health care among women living with HIV.
